# Thickness effect of ultra-thin Ta_2_O_5_ resistance switching layer in 28 nm-diameter memory cell

**DOI:** 10.1038/srep15965

**Published:** 2015-11-03

**Authors:** Tae Hyung Park, Seul Ji Song, Hae Jin Kim, Soo Gil Kim, Suock Chung, Beom Yong Kim, Kee Jeung Lee, Kyung Min Kim, Byung Joon Choi, Cheol Seong Hwang

**Affiliations:** 1 Department of Materials Science and Engineering and Inter-university Semiconductor Research Center, Seoul National University, Seoul 151-744, Korea; 2 SK Hynix Inc., 2091 Gyeongchung-daero Bubal-eub Icheon-si Gyeonggi-do, 467-734, Korea; 3 Hewlett-Packard Laboratories, Hewlett-Packard Company, Palo Alto, California 94304, USA; 4 Department of Materials Science and Engineering, Seoul National University of Science and Technology, Seoul 139-743, Korea

## Abstract

Resistance switching (RS) devices with ultra-thin Ta_2_O_5_ switching layer (0.5–2.0 nm) with a cell diameter of 28 nm were fabricated. The performance of the devices was tested by voltage-driven current—voltage (I-V) sweep and closed-loop pulse switching (CLPS) tests. A Ta layer was placed beneath the Ta_2_O_5_ switching layer to act as an oxygen vacancy reservoir. The device with the smallest Ta_2_O_5_ thickness (0.5 nm) showed normal switching properties with gradual change in resistance in I-V sweep or CLPS and high reliability. By contrast, other devices with higher Ta_2_O_5_ thickness (1.0–2.0 nm) showed abrupt switching with several abnormal behaviours, degraded resistance distribution, especially in high resistance state, and much lower reliability performance. A single conical or hour-glass shaped double conical conducting filament shape was conceived to explain these behavioural differences that depended on the Ta_2_O_5_ switching layer thickness. Loss of oxygen via lateral diffusion to the encapsulating Si_3_N_4_/SiO_2_ layer was suggested as the main degradation mechanism for reliability, and a method to improve reliability was also proposed.

Ta_2_O_5_ has been considered as a useful resistance switching (RS) element in resistance-switching random access memory (RRAM)^1^,^2^,^3^,^4^,^5^. Researchers have observed that Ta_2_O_5_ exhibits mostly bias-polarity-dependent valence change mechanism (VCM) mediated by repeated generation and rupture of conducting filaments (CF), which could be the clusters of oxygen vacancies (V_O_) or Ta atoms^6^,^7^. It was found that RRAM performance of Ta_2_O_5_ layer can be greatly improved if it was placed with a nearby defect reservoir layer, such as Ta or TaO_x_ (x ~ 2)^3^,^4^,^5^,^6^,^7^,^8^. In this case, the oxygen vacancies move back and forth between the local CF region in the Ta_2_O_5_ RS layer and the reservoir layer, with minimal involvement of oxygen loss through the top and bottom electrodes, which could have otherwise degraded endurance^3^.

Kim *et al.*^8^ recently reported that the resistance values of low resistance state (LRS) is almost independent of the Ta_2_O_5_ layer thickness (6.2–9.4 nm), which were generated through oxidation of the sputtered 20 nm-thick TaO_x_ (x ~ 2) reservoir layer by a pulsed-plasma application with different plasma-cycle number, in their self-limited RS mechanism. The thickness of the remaining Ta_2_O_5_ region (other than CF) played crucial role in controlling the switching mode of the self-limited operation scheme, signifying the importance of the RS layer thickness. Many other reports^3^,^4^,^5^,^6^,^7^,^9^ on the RS of Ta_2_O_5_ generally adopted the film thickness of ~ several to several tens of nm. On the other hand, the eventual structure of RRAM, which may succeed the vertical NAND flash after it will stop scaling, will be the vertical RRAM, most probably with vertical placement of bit line and laterally stacked layers of word planes (See Fig. 7 of ref. 10). In this case, the space allowed for the placement of RS layer and cell selectors is far lower than ~5 nm for sub-10 nm design rule devices. Therefore, the Ta_2_O_5_ layer must be much thinner than what many of the previous works have adopted. In this work, according to this constraint, ultra-thin Ta_2_O_5_ layer (0.5, 1.0. 1.5 and 2.0 nm) was deposited by atomic layer deposition (ALD) on a Ta reservoir layer, which was deposited by sputtering. In order to avoid interference of any uncontrolled defect in such an ultra-thin RS layer, the memory cell was fabricated with the deep ultra-violet lithography technology with an active memory cell diameter of 28 nm. The cell architecture was cross-bar array (CBA), with TaN bottom electrode (BE) and TiN top electrode (TE). In this structure, the Ta_2_O_5_ RS layer contacted the TiN TE and the Ta reservoir layer contacted the TaN BE. The small memory cell was surrounded by Si_3_N_4_/SiO_2_ insulator and an external wire was patterned using the back-end-of-the-line process which greatly improved the reliability of the device. This high level of reliability allowed the authors to achieve statistically meaningful data from such a tiny memory cell. The switching performances were checked with conventional current—voltage (I-V) sweep as well as a closed-loop pulse switching (CLPS) mode, where the feedback of each resistance values to the subsequent pulse application precisely controlled the final resistance value to the targeted values.

It was found that there was a critical difference in the switching performance between the thinnest film (0.5 nm) and the other thicker films (1.0–2.0 nm) in terms of the I-V, CLPS. An appropriate switching mechanism based on the hour-glass shaped CF model, regarding the generation and configuration of CF in the ultra-thin Ta_2_O_5_ layer depending on the thickness, was suggested to explain such critical differences.

## Results

### Abrupt electroforming and abnormal RESET behaviour of thicker Ta_2_O_5_ devices

**Figure 1:**
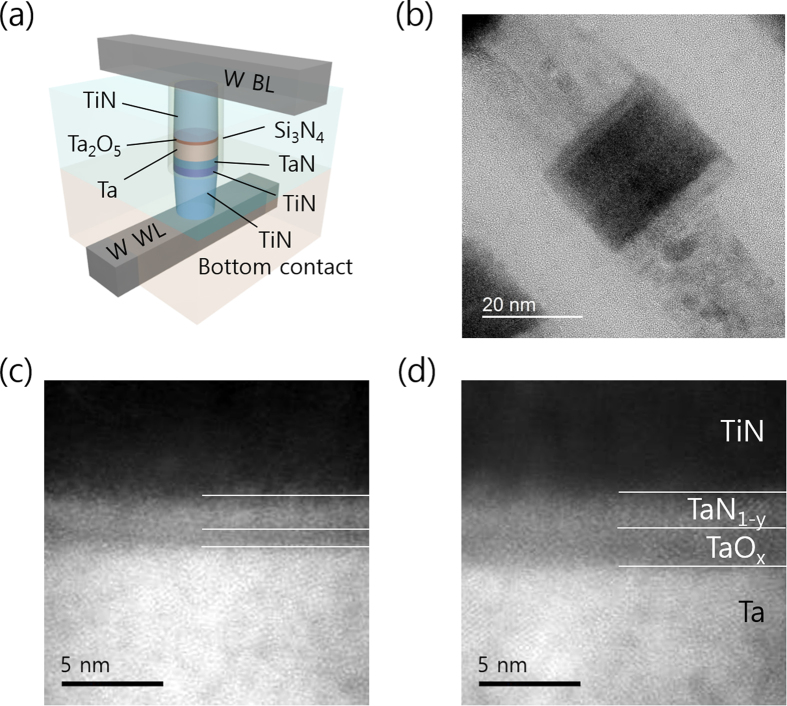
(**a**) Schematic diagram and (**b**) TEM image of the TiN/Ta_2_O_5_/Ta/TaN device. Ta_2_O_5_ (0.5 nm) device, and (**d**) Ta_2_O_5_ (1.5 nm) device. Insets show the linear I-V plot. Scanning transmission electron microscopy (STEM) high angle annular dark field (HAADF) images of (**c**) 0.5 nm-thick device and (**d**) 2.0nm-thick device.

A schematic diagram and transmission electron microscopy (TEM) image of the TiN/Ta_2_O_5_/Ta/TaN device are shown in Fig. 1a,b, respectively. Refer to experimental section for details of the device fabrication process. Figure 1c–d show high angle annular dark field (HAADF) scanning transmission electron microscopy (STEM) images of 0.5 nm-thick device and 2.0 nm-thick device. It was found that the actual thickness of the switching layers in each device was thicker than the targeted value (by controlling ALD cycle number) by a common value of ~0.3 nm, which might be due to the oxidation of bottom Ta reservoir layer. However, for the sake of simplicity, the values from the ALD process will be denoted as Ta_2_O_5_ layer thickness (t_TaO_) in this report. Etched side surfaces of the memory cell were protected by thin Si_3_N_4_ layer (7 nm), which protected the side areas of memory cell from further oxidation.

Pristine TiN/Ta_2_O_5_/Ta/TaN devices usually show a high resistance of ~10^4^–10^6^ Ω, and display typical bipolar resistance switching (BRS) behaviour after the electroforming step. The electroforming and SET (switching from high resistance state (HRS) to LRS) switching were accomplished with negative bias while the RESET (switching from LRS to HRS) switching was achieved with a positive bias. These factors suggested that the RS in this material might be mediated by the generation of V_O_ at the Ta_2_O_5_/Ta and their migration toward the TE interface forming a CF, which will be discussed in detail later.

**Figure 2:**
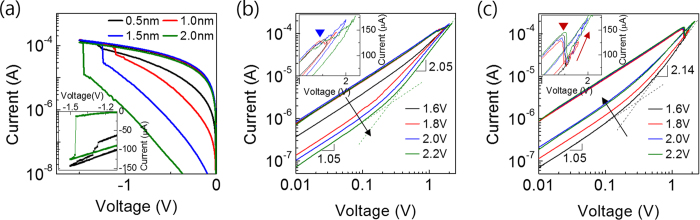
(**a**) Forming and 1^st^ RESET behavior of Ta_2_O_5_/Ta devices. Double-logarithm I-V curves of 1^st^ RESET behavior depending on RESET voltage of (**b**) Ta_2_O_5_ (0.5 nm) device, and (**c**) Ta_2_O_5_ (1.5 nm) device. Insets show the linear I-V plot.

Figure 2a shows the I-V curves of the pristine devices with different t_TaO_ confirming the electroforming behaviour under negative bias polarity. Due to the involvement of a high R_s_ (~8 kohm), no compliance current was settled but all the devices showed a limited maximum current of ~100 μA at −1.0 V. As t_Tao_ value increased the current value generally decreased, which was in accord with a general idea that thicker pristine film is more insulating. The electroforming voltage decreased (absolute value increased) with increasing t_TaO_ for t_TaO_ values of 1.0–2.0 nm. By contrast, the electroforming voltage for t_TaO_ = 0.5 nm decreased (absolute value increased) compared with t_TaO_ = 1.0 nm device. Such peculiar behaviour of t_TaO_ of 0.5 nm device was also due to the involvement of large R_s_, whose details will be reported in other report. From the appropriate modelling of circuitry of the devices, a constant electroforming field of 0.54 V/nm was calculated. It was also found that additional 0.3 nm t_TaO_ to the targeted value is needed for fitting the experimental data, which is in agreement with the value confirmed by TEM images. Detailed discussions on this characteristic feature of electroforming step was reported in our previous study^11^.

Another critical difference between the devices with t_TaO_ of 0.5 nm and others is the rate of current variation at the moment of electroforming. This was clearly observed from the inset I-V curves of the 0.5 nm and 2.0 nm devices, which zoom up the I-V of Fig. 2a near the electroforming stage. The 2.0 nm device was observed to show a very abrupt electroforming process, whereas the 0.5 nm device exhibited a much gradual process. The abrupt electroforming for thicker devices with larger t_TaO_ values suggested that a certain acceleration process of CF formation was involved, such as field concentration due to a sharp geometry of CF or involvement of hour-glass shape CF formation. However, the gradual electroforming for the case of t_TaO_ = 0.5 nm suggested that these effects were negligible. Such critically different switching behaviours of the two groups could also be observed for RESET process under the positive bias region, as shown in Fig. 2b,c, which represent the RESET I-V curves of the 0.5 nm and 1.5 nm devices, respectively.

There are two notable differences between these two graphs. For the case of 0.5 nm device, the current of HRS after RESET generally decreased with increasing RESET voltage (maximum voltage applied during the RESET I-V sweep), which can be easily understood from the more evident rupture of CF with increasing RESET voltage. However, this was not the case for the 1.5 nm thick device, where the increasing RESET voltage actually increased the HRS current, which is in a stark contrast to the results in Fig. 2b. Another difference between these two devices is in their behaviour at the moment of RESET switching, which is evident from the inset figures. The 0.5 nm device showed a gradual decrease in current with increasing voltage after the onset of RESET switching indicated by blue triangle in the inset of Fig. 2b, which is similar to gradual electroforming. It can be also noted that the current in HRS decreased with increase in the maximum voltage after the RESET. By contrast, the 1.5 nm device showed an abrupt decrease in current at the moment of RESET (indicated by the red triangle in inset of Fig. 2c). Another critical difference is that the I-V curves after the onset of RESET had a higher slope than that of LRS, which eventually induced increased HRS current when the maximum voltage after the RESET was increased. This actually corresponded to a kind of partial SET process. Devices with other t_TaO_ values (1.0 and 2.0 nm) showed similar RESET I-V switching behaviours as in Fig. 2c (data not shown).

Electrical conduction mechanism of the devices can be inferred from the slopes of the I-V curves in LRS and HRS in the double logarithmic plots shown in Fig. 2b,c. For LRS in both cases, the slopes were commonly ~1 before the onset of RESET, which suggested that the CF mediated an ohmic behaviour. For the case of HRS, the slopes of I-V curves were ~1 and ~2 for low and high voltage regions, which coincided quite well with the space charge limited current (SCLC) conduction mechanism^12^. This implied that the RESET process ruptured the CF and V_O_-dispersed Ta_2_O_5_ matrix conducted the electricity via the SCLC mechanism.

### Abnormal SET behaviour of thicker Ta_2_O_5_ devices in CLPS measurement

With a view to understanding more precisely these critical differences in the electrical switching behaviours between the thinnest t_TaO_ device and other thicker t_TaO_ devices, CLPS^13^ tests were attempted for the devices shown in Fig. 2b,c.

**Figure 3:**
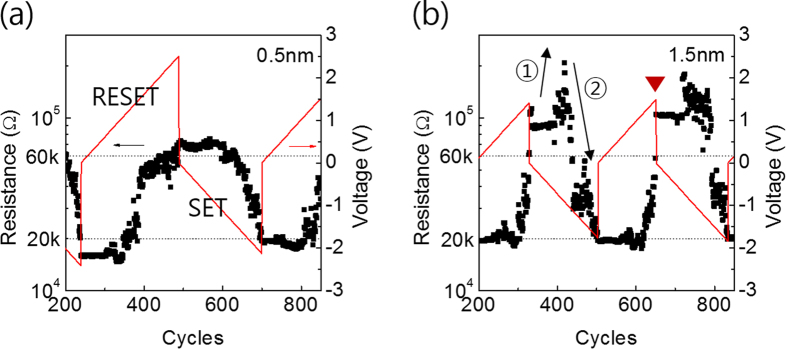
Feedback-loop pulse measurements of (**a**) Ta_2_O_5_ (0.5 nm) device and (**b**) Ta_2_O_5_ (1.5 nm) device. The red lines indicate the write (SET/RESET) voltage and the black dots represent resistance of devices. The dashed line has been added to show the setting values (20 kΩ, 60 kΩ).

In CLPS test, the devices under test (DUT) could be either pristine or the one after the SET or RESET by the I-V sweep. In this report, pristine devices with t_TaO_ of 0.5 nm and 1.5 nm were considered, and the CLPS test results are shown in Fig. 3a,b. For these tests, the target resistance values of LRS and HRS were set at 20 kohm and 60 kohm estimated at 0.2 V, which were sufficiently higher than R_s_ but low enough to confirm a fast reading considering the Johnson-Nyquist limitation^14^ of current read. Since the DUT was pristine, another electroforming was performed in this CLPS test. Figure 3 shows the sequence of RESET and SET behaviours from the LRS of the two DUT. The red line shows the variation of pulse voltages with pulse cycle number, and black dots show the measured resistance values after each pulse measured at 0.2 V.

Since the DUT was in LRS, the CLPS in Fig. 3a started with RESET pulse application (2μs) in a positive bias region. It can be noted that the application of positive pulse with a height up to ~1 V (up to cycle number of ~350) could not induce a RESET switching. However, when it was higher, the resistance increased abruptly and slowly reached the target value of 60 kohm as the RESET pulse height further increased to ~2.5 V (cycle number of 500). This suggested that most of the changes in physical status of the CF during the RESET process in this DUT occurred at the RESET voltage range of 1 V- 1.5 V, although a higher RESET pulse voltage of 2.5 V was necessary to achieve the targeted 60 kohm.

**Figure 4:**
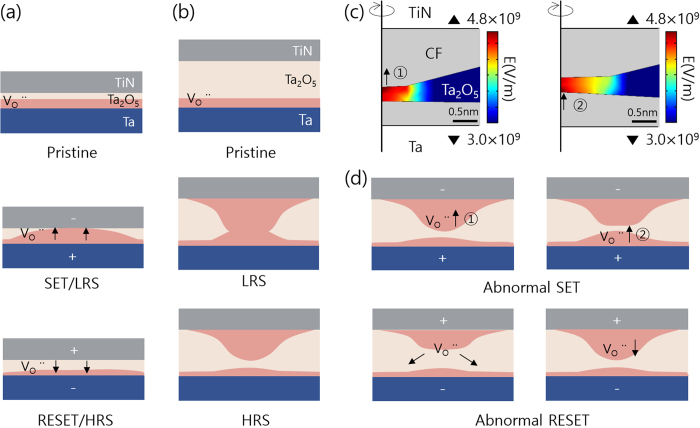
Schematic diagram of the memory cell at the pristine (upper panel), LRS (middle panel) and HRS (lower panel) of the device with t_TaO_ of (**a**) 0.5 nm (**b**) 1.5 nm. (**c**) Electric field distribution, calculated by COMSOL package. (**d**) Schematic diagrams depicting abnormal SET (upper two panels), and abnormal RESET (lower two panels) behaviors.

Since the target RESET resistance was reached, the test sequence was (automatically) switched to SET process starting from 0 V to negative bias values in the subsequent cycles. As for the RESET process, no major changes in the resistance occurred down to a SET pulse voltage of ~−1 V. However, as the SET pulse height further decreased, the resistance decreased rather gradually and the targeted SET resistance of 20 kohm was obtained at ~−2.2 V (~700 cycle). The typical required cycle numbers for the RESET and SET switching were 176 and 202 cycles, respectively. These CLPS behaviours could be readily understood from a model containing the bias-polarity dependent growth and contraction of CF, as shown in Fig. 4. The gradual transitions between the LRS and HRS in this CLPS also coincided with gradual electroforming (and SET too, data not shown) and RESET transitions observed in the I-V curves in Fig. 2a,b.

By contrast, the DUT with t_TaO_ of 1.5 nm (1.0 and 2.0 nm too, data not shown) showed quite different CLPS behaviours, as shown in Fig. 3b. In this case, the RESET started to occur at ~1.0 V. However, the RESET transition was much more abrupt than Fig. 3a, and was completed at ~1.5 V with smaller number of total RESET pulses (149 cycles vs. 251 cycles in Fig. 3a). Such an abrupt transition coincided with the abrupt RESET behaviour, shown in Fig. 2c. The SET behaviour in CLPS test showed even more peculiar characteristics. As indicated by ①, at cycle number ~400, the resistance temporarily increased quite largely even with the negative (SET polarity) pulses, before it actually switched to LRS at ~−1.8 V (indicated by ② in the figure). This meant that the SET process, which is called abnormal SET in this report, involved detailed microscopic processes, which did not induce any straightforward rejuvenation of CF. In order to explain all these different SET and RESET behaviours in I-V sweep and CLPS, the following CF model in the Ta_2_O_5_ layer was conceived, and their motion under the bias field was simulated using a commercial package COMSOL.

## Discussions

Figure 4a shows the schematic diagram of the memory cell at the pristine (upper panel), LRS (middle panel) and HRS (lower panel) of the device with t_TaO_ of 0.5 nm. It was assumed that the Ta/Ta_2_O_5_ interface originally contained a high density of V_O_ by the chemical interaction between the two layers (Ta works as source of V_O_ or sink of O ions) at the pristine state. By applying the positive voltage to the top electrode, V_O_s were pushed away from the TiN TE and the gap between TE and V_O_ containing region widened, which decreased the current even below the value of the pristine state (data not shown). When a negative bias was applied, the volume of V_O_ containing region near the Ta/Ta_2_O_5_ interface increased by further migration of oxygen ions into the Ta layer, and when this defective region touched the TiN TE, the device switched to LRS (middle panel). Upon reversing the bias polarity, this defective region contracted and separated from the TiN TE letting the device return to HRS. During this entire course, the gradual increase and decrease of the defective region according to the bias were naturally conceived, which explained well the gradual SET and RESET behaviours in I-V sweep and CLPS. However, this model could not explain the abrupt and abnormal switching behaviours in devices with thicker t_TaO_. To explain these characteristics, an hour-glass shape CF^15^,^16^,^17^ was conceived, as shown in Fig. 4b. The upper panel shows the schematic diagram of the pristine state, where the defective region was formed near the Ta/Ta_2_O_5_ interface region too via chemical reaction. However, when a negative bias was applied, some of the V_O_ were dragged onto the TiN TE interface forming the upper portion of CF. It was further anticipated that this upper portion of CF might have a conical shape according to the previous reports^18^,^19^,^20^ (middle panel). Under this circumstance, the electric field was concentrated on the protruded part which eventually induced an abrupt switching into the LRS making an hour-glass shaped CF in LRS after the electroforming step. The formation of such conical-shaped upper portion was not expected for the case of 0.5 nm t_TaO_ device because the thickness of Ta_2_O_5_ layer was too small to form such structure (or expansion of lower defective region during the negative bias application was faster than the formation of upper portion of CF). Upon the application of RESET bias, the upper and lower portions of the CF could be separated and the device went back to HRS. It should be noted that there were some remaining CF portions near the lower Ta and TiN TE after the RESET which is a different situation from the pristine state, and it was believed that those remaining portions induced the abnormal behaviours during subsequent switching.

Figure 4c shows the electric field distribution within the memory cell remaining at the HRS when a SET pulse voltage of −1.5 V was applied, calculated by COMSOL. For this simulation the electrical conductivity of CF regions (indicated by grey colour in the figure) was assumed to be 100 times higher than the remaining insulating region, where the field distribution was calculated. Due to the more conical configuration of the upper portion of residual CF, the electric field was concentrated near the tip region of upper CF portion (indicated by arrow ① in left panel of Fig. 4c). Under this circumstance, movement of the interface between the upper CF portion and the insulating region toward the TE direction was faster than that of the interface between the lower CF portion and the insulating region with a flat shape. Therefore, the gap between the two portions actually increased even though a SET bias (positive bias to TE, which was supposed to decrease the gap) was applied. As this process continued, the upper interface became less conical, whereas the lower interface became more conical (indicated by arrow ② in right panel of Fig. 4c), which then decelerated and accelerated the motions of upper and lower interfaces, respectively. With this two-step process, the abnormal SET in CLPS experiment can be explained. This two-step abnormal SET process is schematically described again by two upper panels of Fig. 4d. For the RESET switching, there was hardly such two-step process because the hour-glass shaped CF penetrated through the entire thickness and the rupture of the weakest part (near the contact between the upper and lower portion of CF) separated the two portions in a one-step process at the moment of RESET. Furthermore, the more curved nature of the lower interface in the LRS induced faster movement of it compared with the upper interface, which had a flatter shape upon the RESET bias application (positive bias to TE). Once this separation occurred, further movement of the lower interface was accelerated because the interface involved insulating region, making the RESET highly abrupt. In CLPS experiment, because of the rapid increase in cell resistance after separation, the test circuit instantaneously switched to the SET mode. However, in I-V sweep, the voltage was further increased even after the actual RESET occurred (Fig. 2c). During this additional application of RESET voltages at higher voltage region, the lower interface became flat (as for the starting point of SET switching), and its movement almost stopped. However, the upper interface started to become curved and it moved toward the BE. With further increase in the maximum RESET voltage in I-V sweep, this increased curving of the upper interface made the cell resistance smaller even though it still remained in the HRS. This model explained satisfactorily the increase in the HRS current with increasing RESET voltage in Fig. 2c. Similar process cannot be expected from the RESET process involving only lower portion of the CF in the case of 0.5 nm device. Lower two panels of Fig. 4d shows the schematically showed the abnormal RESET processes for the thicker Ta_2_O_5_ devices. The switching for the thicker Ta_2_O_5_ device involved a much more complicated process (the insulating region between the two portions of CF bended up and down in addition to its variation in thickness according to the switching cycle) compared with the simpler and straightforward process in thinner Ta_2_O_5_ device. Such complication may induce reliability concern, as discussed next.

**Figure 5:**
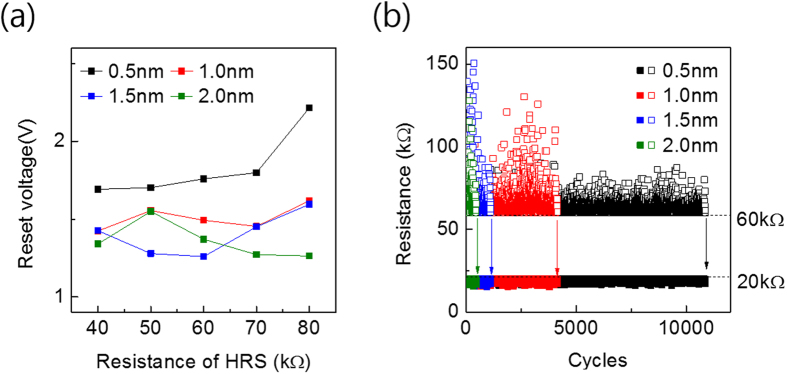
(**a**) Reset voltage for TiN/Ta_2_O_5_/Ta/TaN devices as a function of HRS setting value in feedback-loop measurements. (**b**) Endurance cycles of feedback-loop measurements with LRS 20 kΩ, HRS 60 kΩ settings.

Figure 5a shows the variation in RESET voltage during the CLPS as a function of settled target resistance of HRS for the four devices with different t_TaO_ values. The reset voltage was defined as the voltage when target resistance of HRS was reached. For 0.5 nm t_TaO_, the reset voltage consistently increased with increasing target resistance of HRS, which coincided well with the simple CF configuration in Fig. 4a; to induce a wider the gap, a higher reset voltage was needed. By contrast, the other three devices showed essentially no evident tendency in their reset voltages for the different target resistance of HRS. This can be understood from the very abrupt behaviour of RESET, meaning that the resistance value at the moment of RESET was not well controlled. This behaviour was favourably correlated with the endurance test using the CLPS test as shown in Fig. 5b.

Figure 5b reveals that the maximum endurance cycle of the 0.5 nm device was as high as ~12,000 with a narrower distribution in the HRS resistance value. However, with the increasing t_TaO_, the endurance cycle drastically decreased and became only ~1,000 for the 2.0 nm device. The distribution of resistance value of HRS became much wider as the t_TaO_ increased. Once the devices failed, they mostly showed a SET-stuck behaviour, i.e. it could not be RESET anymore. On the other hand, the resistance values of LRS were much more uniform, which can be ascribed to the presence of R_s_; it prohibited excessive current flow when the device SETs. The variation in resistance value of HRS even in CLPS test can be explained from the resistance profile shown in Fig. 3b. For example, at the moment of RESET at ~650 cycle (indicated by red down triangle in the figure) the resistance suddenly jumped from a value slightly lower than 60 kohm (so the test circuit applied another RESET pulse with slightly higher amplitude) to ~100 kohm. Then, the test circuit started to send SET pulses subsequently after recording the HRS resistance of ~100kohm for this pulse sequence. If the RESET pulse length was shorter than the present value (2 μs), then such an abrupt and uncontrolled RESET might have been controlled better, but 2 μs was the minimum pulse length that this test circuit could produce. Nevertheless, relative comparison of the distribution of RESET resistance among the different devices was important in the estimation of the device performance.

The limited endurance and general SET-stuck behaviours of the devices with thicker t_TaO_ can explain an endurance degradation mechanism as follows. When the device was RESET excessively, i.e. RESET resistance was much higher than the target value, the gap between the two portions of the CF became wider than that with the well-controlled HRS resistance value. The factor that further aggravated the circumstance was the temporary RESET during subsequent SET pulsing. Then, the subsequent SET required consumption of higher SET power to change the resistance state. Overall, the abrupt SET/RESET processes of the thicker t_TaO_ devices involved larger consumption of power, which induced significant Joule heating effect. It could be assumed that the Joule heating effect induced lateral motion of oxygen atoms to the nearby passivating oxide layer^1^,^21^, which in turn induced endurance degradation and SET-stuck. Although such lateral diffusion was less severe for 0.5 nm device, as can be understood from the higher endurance cycle in Fig. 5b, there could be also involvement of Joule heating effect albeit it was less significant. It could be anticipated that encapsulating the memory cell with oxygen-migration blocking layer, such as Si_3_N_4_, could be a feasible method to suppress such reliability concern.

This work can be compared with the recent report by Chen *et al.*^9^. In that report, they examined the endurance degradation mechanisms from a memory cell whose structure was very similar with the current one; TiN/Ta_2_O_5_/Ta, where the Ta_2_O_5_ layer was grown by ALD with a thickness of 6 nm, and Ta acted as a V_O_ reservoir. They also determined that the loss of oxygen during the repeated switching cycles was the main endurance degradation mechanism, but the major difference from this work is that the loss of oxygen was ascribed to the diffusion of oxygen into the TiN electrode. Depending on the RESET pulse conditions, they found the SET-stuck or RESET-stuck behaviours (severe RESET pulse induced RESET-stuck). Since this work has not found any RESET-stuck problem irrespective of the device condition, the failure mechanism could be different from that reported by Chen *et al.* It was not clearly stated if their memory cell, which had a simple cross-bar structure with an area of 100 × 100 nm^2^, was passivated with SiO_2_ layer or not. The smaller cell size in this work, which increased the chance of lateral diffusion of oxygen toward the passivation layer, could be one of the reasons for different endurance degradation mechanism. Their endurance cycle was (10^6^–10^8^) generally much higher than the present work (10^4^). This could be due to the much longer pulse time in this work (2 μs) vs. the previous work (1–10 ns). In their case, the longer pulse time (1 μs) also decreased the endurance cycle from 10^8^ (for 1 ns) to 10^6^ cycles. The smaller thickness of the Ta_2_O_5_ layer in the present work could have been unfavourable for ensuring a large endurance cycles. This was because the smaller thickness Ta_2_O_5_ contained a lower number of oxygen ions, even for the optimized cycling condition (SET/RESET pulse heights and durations). This is another critical parameter that needs to be considered for reliable RRAM fabrication. Nonetheless, other undesirable behaviours of the CF during their formation, rupture (disintegration into the upper and lower portions) and reconnection in thicker films overwhelmed the merit of higher number of oxygen ions in the memory cell.

In conclusion, RS characteristics of RRAM device with Ta/Ta_2_O_5_ stacked RS layers with ultra-thin Ta_2_O_5_ thicknesses (0.5–2.0 nm) were examined by the voltage-driven I-V sweep and closed-loop pulse switching test. While the device with the smallest Ta_2_O_5_ thickness showed normal switching properties with a gradual change in resistance and high reliability in voltage sweep or pulse switching, other devices with larger Ta_2_O_5_ thickness showed several abnormal behaviours with respect to the detailed SET and RESET processes, degraded resistance distribution, especially in HRS, and much lower reliability performance. These critical differences could be explained by the one sided CF model for the former case, where the gradual expansion and contraction of it are responsible for the reliable RS, and double sided hour-glass shaped CF model for the latter case, which involved much complicated evolution of CF shape and many abnormal behaviours. The involvement of abnormal SET and over-RESET behaviours, which were related with the up and down buckling motion of the insulating region for the devices with thicker t_TaO_ RS layer, was accompanied with excessive Joule heating effect. This effect inevitably increased the power consumption as well as the reliability concern, which could be reasonably ascribed to the lateral diffusion of oxygen to the nearby Si_3_N_4_/SiO_2_ passivating layer, which could be facilitated by the small thickness (7 nm) of Si_3_N_4_ layer. Encapsulating the device with thicker oxygen impermeable layer was expected to improve the reliability.

## Methods

The memory cell was fabricated as follows. After patterning a W layer for the word lines, SiO_2_ layer was deposited by chemical vapour deposition (CVD) method. Then, contact hole to the W bit line was made within the CVD SiO_2_ and filled with TiN layer. After chemical mechanical polishing of the TiN layer to isolate the contacts, TaN BE layers and a Ta layer was deposited by sputtering, followed by sequential formation of Ta_2_O_5_ layers with different thickness by ALD at 280^o^ C and a TiN TE layer through reactive sputtering. The TiN/Ta_2_O_5_/Ta/TaN stacked layers were dry-etched with a cell diameter of 28 nm. Furthermore, the fabricated structure was passivated with 7 nm-thick CVD Si_3_N_4_ spacer, and SiO_2_ interlayer dielectric layer was deposited. TE contact was achieved via W bit lines, which ran in orthogonal direction to the word line direction. Al pads were used to contact the bit and word lines. The estimated sum of the resistances of the bit line, contact, and word line per cell was ~8 kohm, which played a role as the series resistance allowing the RS in I-V sweep without setting any compliance current.

The fabricated device structure was confirmed by TEM, and TEM sample preparation was performed by focussed-ion beam etching technique. I-V sweep was performed using a Hewlett Packard 4155B semiconductor parameter analyser with TE (word line) biased and BE (bit line) grounded. Pulse switching in closed loop configuration was performed by an in-house built field programmable gate array board, which allowed a minimum pulse width of 2 μs. This setup allowed repeated pulse application with intermittent reading of resistance value at 0.2 V after each SET or RESET pulse application.

## Additional Information

**How to cite this article**: Hyung Park, T. *et al.* Thickness effect of ultra-thin Ta_2_O_5_ resistance switching layer in 28 nm-diameter memory cell. *Sci. Rep.* **5**, 15965; doi: 10.1038/srep15965 (2015).
